# Momentum-space nonsymmorphic symmetry meets higher-order topology

**DOI:** 10.1093/nsr/nwaf237

**Published:** 2025-06-09

**Authors:** Y X Zhao

**Affiliations:** Department of Physics and HK Institute of Quantum Science & Technology, The University of Hong Kong, China

## Abstract

Momentum-space nonsymmorphic group *Pnnn* has been achieved by alternating 0 and π fluxes in artificial crystals, enabling 3D octupole topological insulators with corner states.

The beauty of crystals in physics is characterized by their crystal symmetries, such as reflections and rotations [1]. These symmetries follow a dichotomy: they are either symmorphic or nonsymmorphic, depending on the existence of invariant points under the symmetry. Crystal symmetries are not only visually embodied in real space, but also exist within reciprocal lattices in momentum space. In conventional crystal-symmetry theory, these symmetries, when operating in momentum space, preserve the origin of this space and hence are categorized as symmorphic symmetry.

Recently, it was proposed that, within a broader framework of projective crystal symmetry, crystal symmetries could be nonsymmorphic in momentum space [2,3]. A basic example, as illustrated in Fig. 1a, is the momentum-space glide reflection, ${G}_x:( {{k}_x,{k}_y} ) \mapsto ( { - {k}_x,{k}_y + \pi } )$, in two dimensions, which occurs when the real-space mirror reflection ${M}_x$ anticommutes with the primitive lattice translation along the *y* direction [3]. As nonsymmorphic symmetry, it can reduce the topology of the momentum-space unit from a torus to a Klein bottle. In their recent work, Qiu *et al.* expanded this 2D glide reflection to three dimensions, resulting in three glide reflections, for example, ${G}_x:( {{k}_x,{k}_y,{k}_z} ) \mapsto ( { - {k}_x,{k}_y + \pi ,{k}_z + \pi } )$, with ${G}_y$ and ${G}_z$ operating similarly [4]. According to the general theory of momentum-space nonsymmorphic groups [2], the three glide reflections in the three orthogonal directions, together with the three primitive reciprocal lattice translations, generate the momentum-space crystallographic group $Pnnn$. Here, $Pnnn$ consists of all combined spatial transformations involving the three glide reflections and the three reciprocal lattice translations.

**Figure 1.**
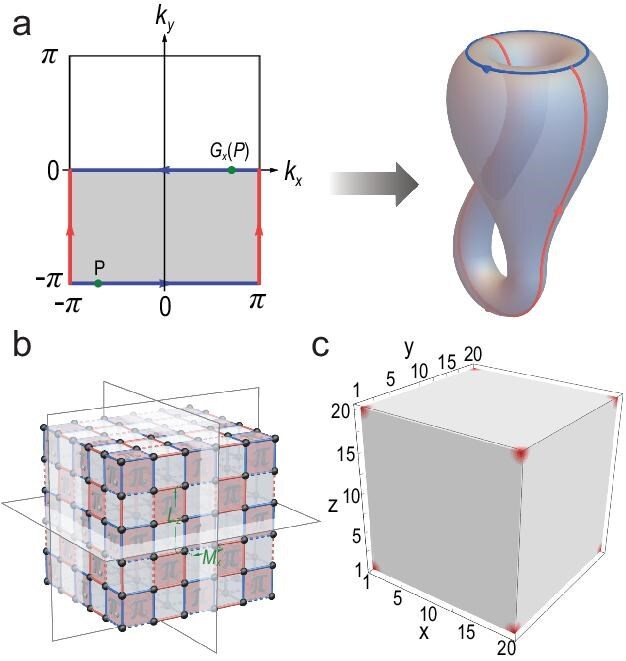
Gauge flux enabled momentum-space glide reflections and the corner states of the symmetry-protected octupole insulator. (a) Momentum-space glide reflection and the induced Brillouin Klein bottle. Point *p* is mapped to ${G}_x( p )$. The glide reflection reduces the Brillouin zone to the shadowed half with boundary gluing rules. Gluing the oriented red and blue edges, respectively, results in the Klein bottle. Adapted from [3] with permission. (b) Flux configuration for realizing three glide reflections in momentum space. The three mirror planes are indicated and red plaquettes host $\pi $ flux, while gray plaquettes host 0 flux. For instance, the rectangle spanned by the mirror reflection ${M}_x$ and the translation ${L}_z$ has flux $\pi $ and therefore ${M}_x$ is represented by ${G}_x$ that involves fractional translation ${G}_z/2$. Dashed (solid) lines indicate negative (positive) hopping amplitudes and hopping magnitudes are distinguished by red and blue colors. Hopping magnitudes are distinguished by red and blue. Adapted from [4] with permission. (c) Corner states of the octupole topological insulator. Adapted from [4] with permission.

On the other hand, the symmetry-protected topological phase has become a central theme in condensed matter in recent years. Naturally, the question arises: what novel topological phases can this new symmetry genre protect? Qiu *et al.*’s work is the first to realize the octupole topological insulating phase protected by momentum-space nonsymmorphic symmetry [4]. This represents a third-order topological insulator with a quantized octupole moment in the bulk, leading to corner in-gap states for cubic geometry (see Fig. 1c).

To implement projective crystal symmetry leading to momentum-space nonsymmorphic symmetry, one can harness the versatility of various artificial crystals to devise suitable gauge-flux configurations across lattices [5]. Importantly, $0$ and $\pi $ fluxes are adequate for achieving momentum-space glide reflections. Introducing a $\pi $ flux through the minimal area generated by a mirror reflection and a lattice translation along the mirror plane in real space enables the momentum-space glide reflection [3]. Qiu *et al.* skillfully engineered the $0 - \pi $ flux configuration (see Fig. 1b) to integrate the three glide reflections across three directions by using periodic arrays of electric circuits as the platform to materialize the octupole topological insulator protected by these momentum-space glide reflections [4].

The work of Qiu *et al.* illuminates that investigating novel topological phases protected by momentum-space nonsymmorphic symmetry, or, more broadly, by projective crystal symmetry, is an exhilarating new frontier in the domain of symmetry-protected topological phases. Notably, the flexibility offered by various artificial crystals in engineering flux configurations renders them advantageous over traditional condensed matter systems for hosting intricate projective symmetry algebras and complex topological structures.
